# A quick and efficient hydroponic potato infection method for evaluating potato resistance and *Ralstonia solanacearum* virulence

**DOI:** 10.1186/s13007-019-0530-9

**Published:** 2019-11-30

**Authors:** Huijuan Wang, Jinxue Hu, Yao Lu, Mancang Zhang, Ning Qin, Ruize Zhang, Yizhe He, Dongdong Wang, Yue Chen, Cuizhu Zhao, Núria S. Coll, Marc Valls, Qin Chen, Haibin Lu

**Affiliations:** 1 College of Agronomy and State Key Laboratory of Crop Stress Biology for Arid Areas, Northwest A&F University, Yangling, 712100 Shaanxi China; 2 Centre for Research in Agricultural Genomics (CRAG), CSIC-IRTA-UAB-UB, Campus UAB, Bellaterra, 08193 Barcelona, Catalonia Spain; 3 Department of Genetics, University of Barcelona, 08028 Barcelona, Catalonia Spain; 4 State Key Laboratory of Crop Stress Biology for Arid Areas, College of Food Science and Engineering, Northwest A & F University, Yangling, 712100 Shaanxi China

**Keywords:** *Ralstonia solanacearum*, Potato, In vitro infection, Brown rot, Bacterial wilt

## Abstract

**Background:**

Potato, the third most important crop worldwide, plays a critical role in human food security. Brown rot, one of the most destructive potato diseases caused by *Ralstonia solanacearum*, results in huge economic losses every year. A quick, stable, low cost and high throughout method is required to meet the demands of identification of germplasm resistance to bacterial wilt in potato breeding programs.

**Results:**

Here we present a novel *R. solanacearum* hydroponic infection assay on potato plants grown in vitro. Through testing wilt symptom appearance and bacterial colonization in aerial part of plants, we found that the optimum conditions for in vitro potato infection were using an OD_600_ 0.01 bacterial solution suspended with tap water for infection, broken potato roots and an open container. Infection using *R. solanacearum* strains with differential degree of aggressivity demonstrated that this infection system is equally efficient as soil-drench inoculation for assessment of *R. solanacearum* virulence on potato. A small-scale assessment of 32 potato germplasms identified three varieties highly resistant to the pathogen, which indicates this infection system is a useful method for high-throughout screening of potato germplasm for resistance. Furthermore, we demonstrate the utility of a strain carrying luminescence to easily quantify bacterial colonization and the detection of latent infections in hydroponic conditions, which can be efficiently used in potato breeding programs.

**Conclusions:**

We have established a quick and efficient in vitro potato infection system, which may facilitate breeding for new potato cultivars with high resistance to *R. solanacearum*.

## Background

*Ralstonia solanacearum* is the causal agent of bacterial wilt (also known as brown rot on potato), one of the most destructive plant diseases on many crops in tropical and subtropical areas, which leads to huge losses in food production [1]. This soil-borne bacterium enters root through wounds or natural openings and multiplies in the vascular tissues, which results in xylem dysfunction and ultimately kills the plant host [2, 3]. The bacterium is also able to survive for years in soil and water retaining the ability to invade host plants [4].

*R. solanacearum* is a heterogeneous species composed of many genetic groups referred to as the *R. solanacearum* species complex (RSSC) [2, 3]. Phylogenetic analyses of several conserved genes revealed that the RSSC is divided into four phylotypes: phylotype I isolates are mainly from Asia, phylotypes II and III are, respectively, composed of American and African strains and phylotype IV strains originate from Indonesia, Japan, Australia and the Philippines [5, 6].

Besides its worldwide geographic distribution, *R. solanacearum* possesses an extraordinarily broad host range, causing disease on more than 200 plant species from 50 different botanical families [3]. The pathogen not only infects solanaceous crops such as tomato, eggplant, peanut, pepper and potato, but also other plants from both the dicot and monocot families, and new hosts are being discovered continuously [7]. Due to its wide geographical distribution, broad host range, long persistence in soil and highly aggressivity on plants, the bacterium was ranked by scholars as the second most important bacterial plant pathogen [1].

Potato is currently the third most important staple food crop for human direct consumption just after rice and wheat and it ranks first in energy and protein production per unit of water [8, 9]. In protein produced per acre land, potato ranks second to soybean. Importantly, potato is rich in microelements and vitamins essential for the human diet, such as vitamin C, potassium and in fiber [8]. Brown rot caused by *R. solanacearum* is one of the most notorious potato diseases, estimated to result each year in 1 billion US$ economic losses worldwide [1]. Breeding new potato cultivars with resistance to brown rot is essential for integrated management of this disease. To this end, the development of procedures to facilitate the screening for resistance in germplasm from wild potato collections, progenies from crosses between potato species and potato transgenic lines will help potato breeding programs.

Soil-drench and stem-puncture inoculation are the two methods most widely used to study plant resistance to *R. solanacearum* [10–13]. However, adult full-size plants are needed in these procedures with the ensuing use of space, energy and time. Furthermore, since *R. solanacearum* is a soil-borne root pathogen, stem penetration bypasses potential root resistance mechanisms. The disadvantage of soil-drench inoculation is that the opacity of soil hinders the direct investigation of root responses to the pathogen. Vass et al. inoculated tomato plants grown in hydroponic conditions to study *R. solanacearum* root colonization [14]. More recently, in vitro pathogenicity assays have been successfully established on tomato [15], Arabidopsis [16, 17], petunia [18] and *M. truncatula* [19]. However, miniaturized in vitro infection assays have not been set up to screen for resistance in potato to the pathogen. We previously generated constitutively luminescent *R. solanacearum* reporter strains, a tool that we have used to characterize the colonization and the defense responses of potato breeding lines [20, 21]. Here we have established a faster, highly efficient, low-cost potato hydroponic infection method to study *R. solanacearum-*potato interactions. Using this new method, we successfully characterized the virulence of several *R. solanacearum* strains on potato and screened for potato varieties showing resistance to *R. solanacearum*. We also simplified the screening process using a luminescent pathogen that can be tracked in vivo in infected plants. This new method will promote the study of potato resistance to *R. solanacearum* and provide insights for investigating other root pathogens of potato under gnotobiotic conditions.

## Results

### Development of an in vitro potato infection system for *R. solanacearum*

**Fig. 1:**
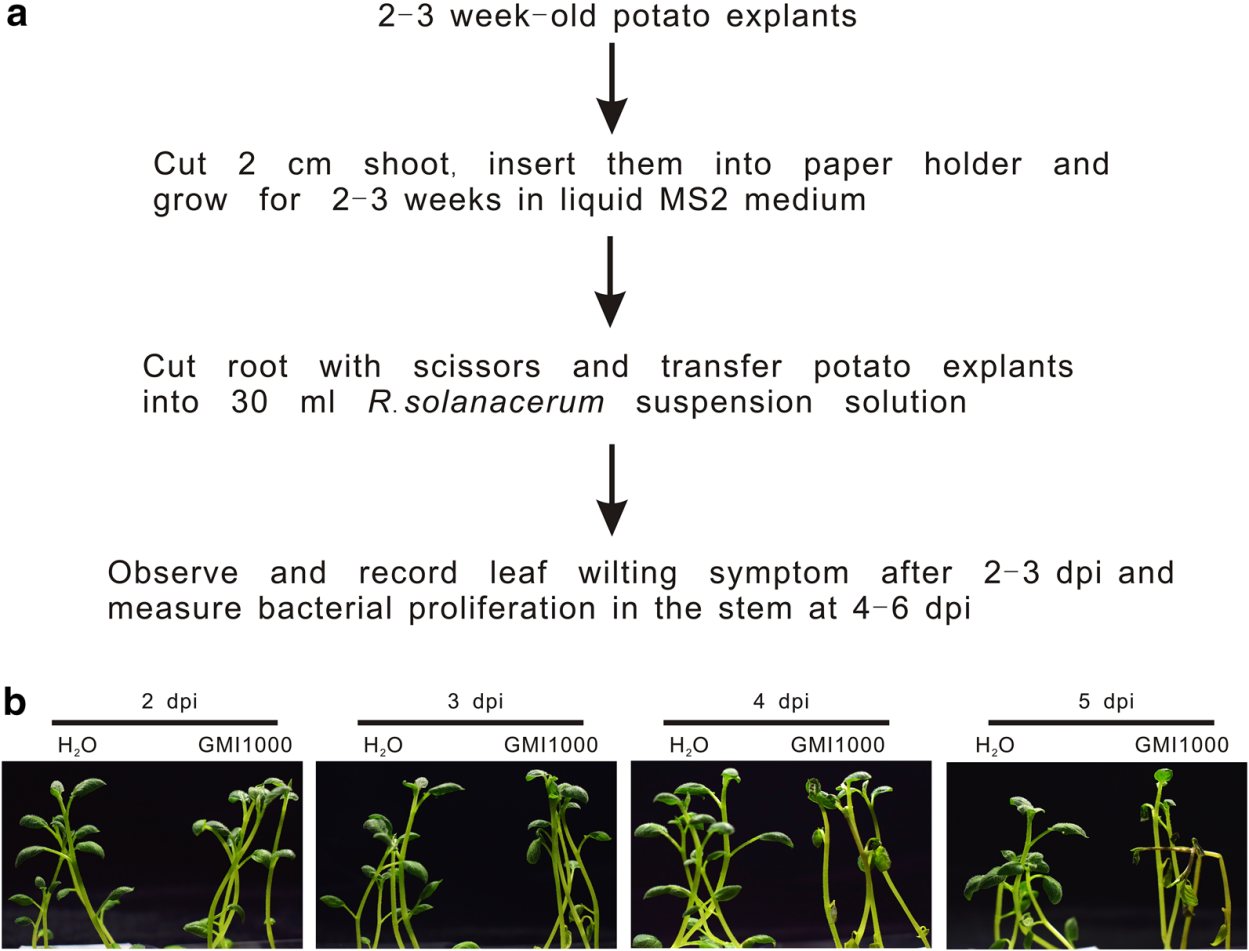
*R. solanacearum* inoculation causes wilting of potato grown under hydroponic conditions. Roots of 2-week-old potato plants were cut and inoculated with 1 × 10^8^ cfu/ml GMI1000. **a** Workflow of the in vitro potato infection method. **b** Representative digital images of bacterial wilt development taken at the indicated days post-infection (dpi). The experiment has been repeated more than three times with similar results

Aiming at the quick identification of potato resistance to *R. solanacearum*, we designed a method for infection in vitro Fig. 1a). A 10^7^–10^8^ colony forming units (cfu)/ml *R. solanacearum* suspension is used to infect plant hosts such as Arabidopsis, tomato and potato [13, 20, 22]. Thus, we grew potato plants hydroponically in MS liquid medium for two weeks, injured the roots and transferred them to the same medium containing 1 × 10^8^ cfu/ml of *R. solanacearum* strain GMI1000, belonging to the phylotype I. The leaves of the infected plants started wilting at 4 days post inoculation (dpi). At 5 dpi, almost all plants exhibited severe wilting symptoms, while the leaves of plants mock-treated with water kept green and healthy (Fig. 1b). This suggested that in vitro infection could be employed to test the virulence of *R. solanacearum* in potato.

**Fig. 2:**
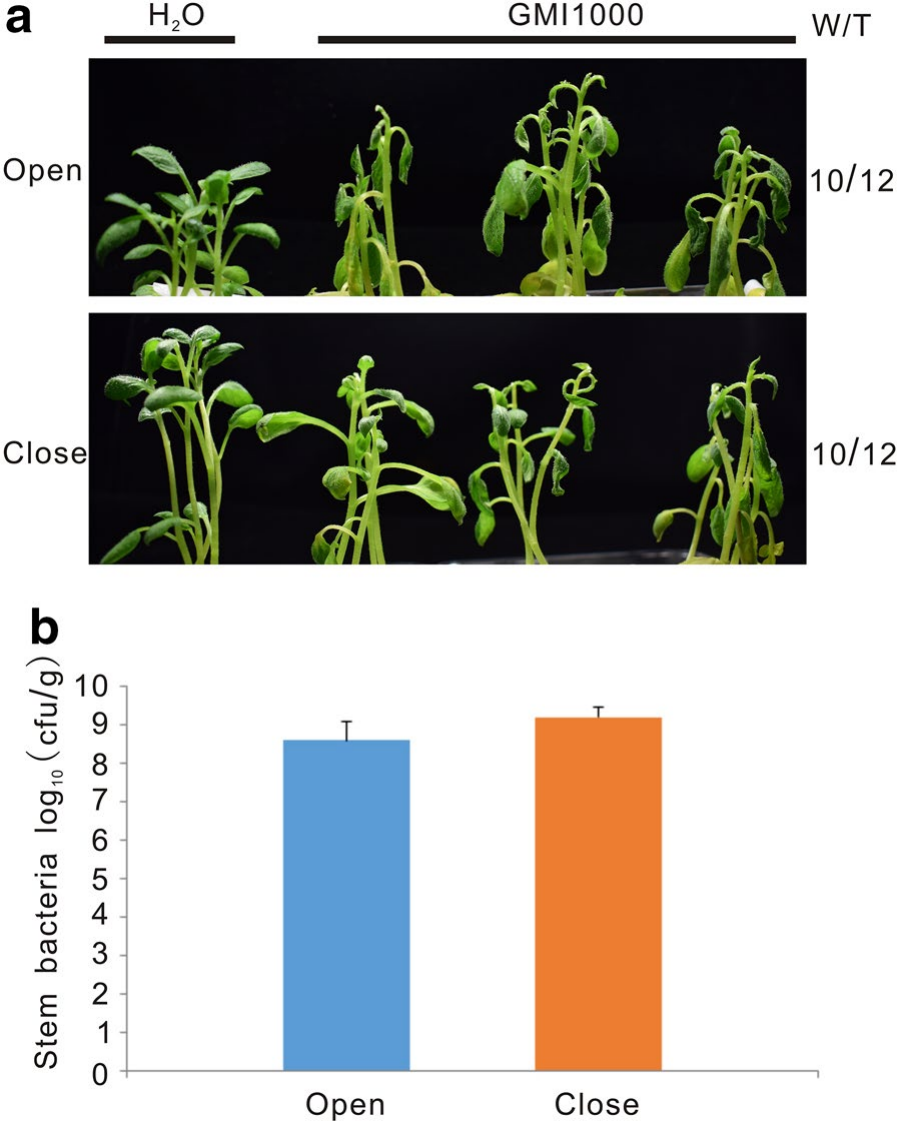
Increased evapotranspiration promoted leaf wilting development on potato plants. **a** Representative plant pictures were taken at 4 dpi. “W/T” represents the number of wilted plants with respect to the total number of infected plants. “Open” indicates air exchange conditions while “close” indicates no air exchange conditions. **b** Bacterial proliferation in potato stems was counted at 4 dpi. Roots of two-week-old potato plants were cut and then inoculated with 1 × 10^8^ cfu/ml of *R. solanacearum* strain GMI1000. The experiment was repeated twice using 12 plants in each experiment

**Fig. 3:**
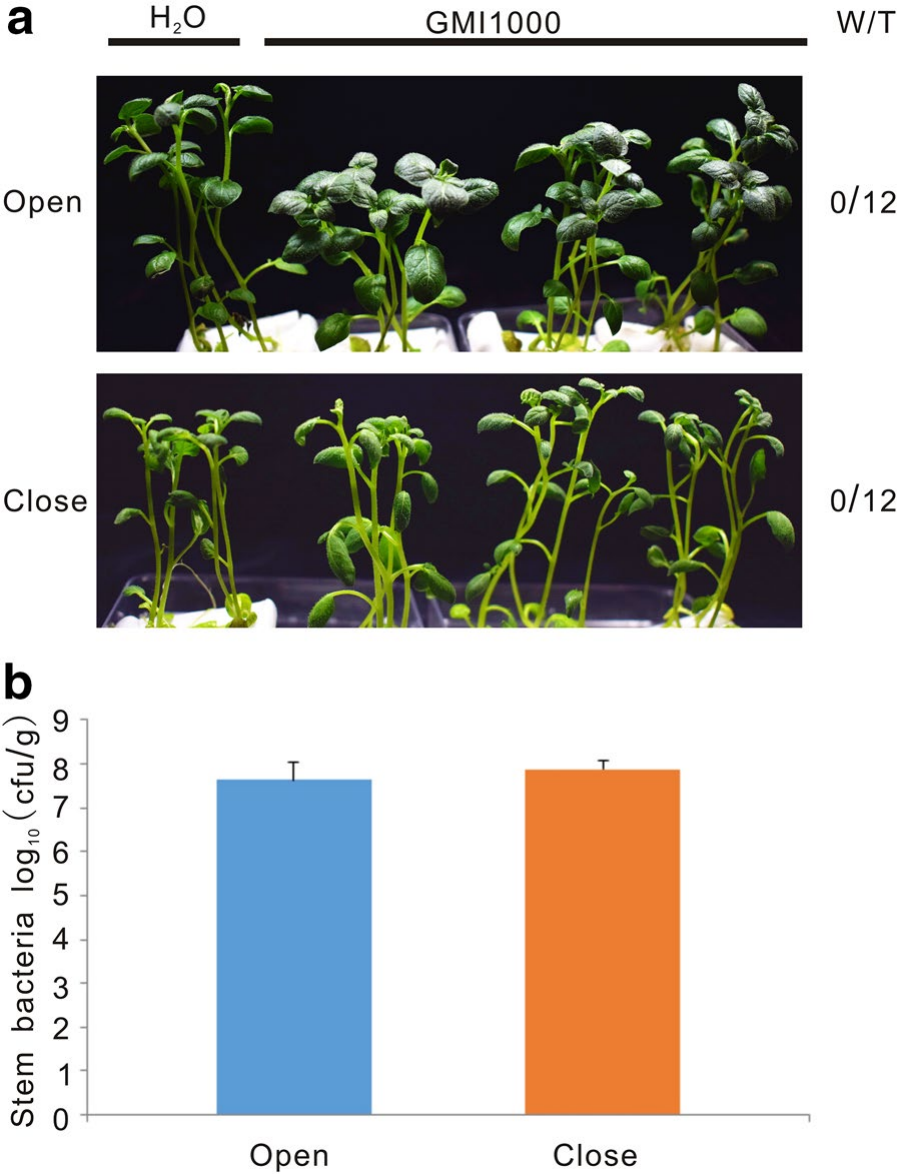
Higher evapotranspiration does not accelerate wilting symptom appearance on plants with intact roots. **a** Representative pictures taken at 9 dpi. “W/T” represents the number of wilting plants with respect to the total of infected plants. “Open” indicates air exchange conditions while “close” indicates no air exchange conditions. **b** Bacterial colonization in potato stems detected at 9 dpi. Two-week-old potato plants were inoculated with 1 × 10^8^ cfu/ml of *R. solanacearum* strain GMI1000 without wounding the roots. The experiment was repeated twice using 12 plants for each

Wilt symptoms appear when water transport in the xylem is blocked by lipopolysaccharides produced by *R. solanacearum* [2]. We thus hypothesized that increased evapotranspiration by opening the growth container’s lids could accelerate plant wilting. To test this possibility, plant inoculations were carried out in parallel under opened and closed lid conditions. Although the same number of wilted plants was observed in both conditions, plants in the open jars exhibited more severe wilting symptoms than those in the close jars (Fig. 2a). We further measured *R. solanacearum* growth in the aerial part of potato plants. The amount of bacteria in plants in the open and close jars was comparable (Fig. 2b), suggesting the air exchange does not affect bacterial growth, but enhances wilting. In nature, *R. solanacearum* enters root at the emergence sites of lateral roots or root tips [2]. To test whether this natural infection also worked in our in vitro infection system, 2-week-old plants without injury were directly infected with the 1 × 10^8^ cfu/ml *R. solanacearum* solution. Even at 9 dpi, no potato plant exhibited wilting symptoms in these conditions (Fig. 3a). To confirm successful colonization in plants, we measured bacterial loads in the aerial plant tissues. The amount of the pathogen in both conditions reached 10^8^ cfu/g at 9 dpi (Fig. 3b), which is 10-fold lower than the 10^9^ cfu/g attained in the root-cut plants at 5 dpi (Fig. 2b). Therefore, plant inoculation without root injury resulted in symptomless infections due to lower bacterial numbers. Thus, the root-cutting treatment was selected for the following experiments based on its faster, reproducible infection, which correlated with the development of wilting symptoms.

**Fig. 4:**
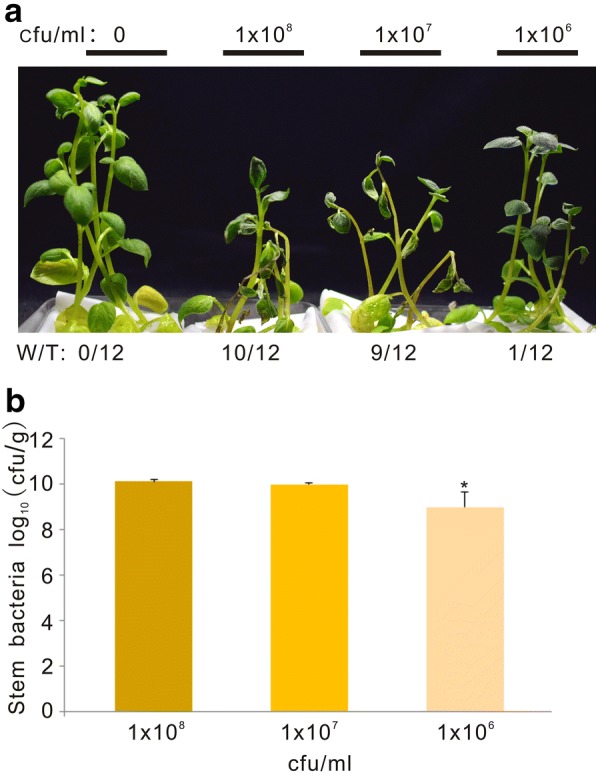
Potato plants infected with different *R. solanacearum* concentrations. **a** Representative pictures taken at 8 dpi. “W/T” represents the number of wilting plants with respect to the total of infected plants. **b** Bacterial colonization in potato stems counted at 8 dpi. The root-cut potato plants were infected with different concentrations of bacterial solutions as indicated. Each experiment was repeated three times using 12 plants each with similar results. Asterisk indicates P < 0.01 (Student’s *t* test) with respect to the 1 × 10^8^ cfu/ml bacteria innocula

The bacterial concentration (1 × 10^8^ cfu/ml) used in laboratory inoculations is usually far higher than that occurring in nature [13, 20, 22]. To check whether lower inocula could be used for in vitro potato infection, disease development in plants was investigated using a series of bacterial suspensions. Eighty percent of plants infected with the higher bacterial solution concentration (1 × 10^8^ cfu/ml and 1 × 10^7^ cfu/ml) showed wilting symptoms at 8 dpi. At this time those plants infected with the lowest bacterial concentration (1 × 10^6^ cfu/ml) just started wilting (Fig. 4a). Moreover, potato plants inoculated with 1 × 10^8^ or 1 × 10^7^ cfu/ml showed similar amounts of bacteria in the aerial part of plant, while those inoculated with the 1 × 10^6^ cfu/ml solution contained fivefold less bacteria (Fig. 4b). This suggested that 1 × 10^7^ cfu/ml is the optimum *R. solanacearum* concentration to inoculate potato plants in hydroponic conditions.

**Fig. 5:**
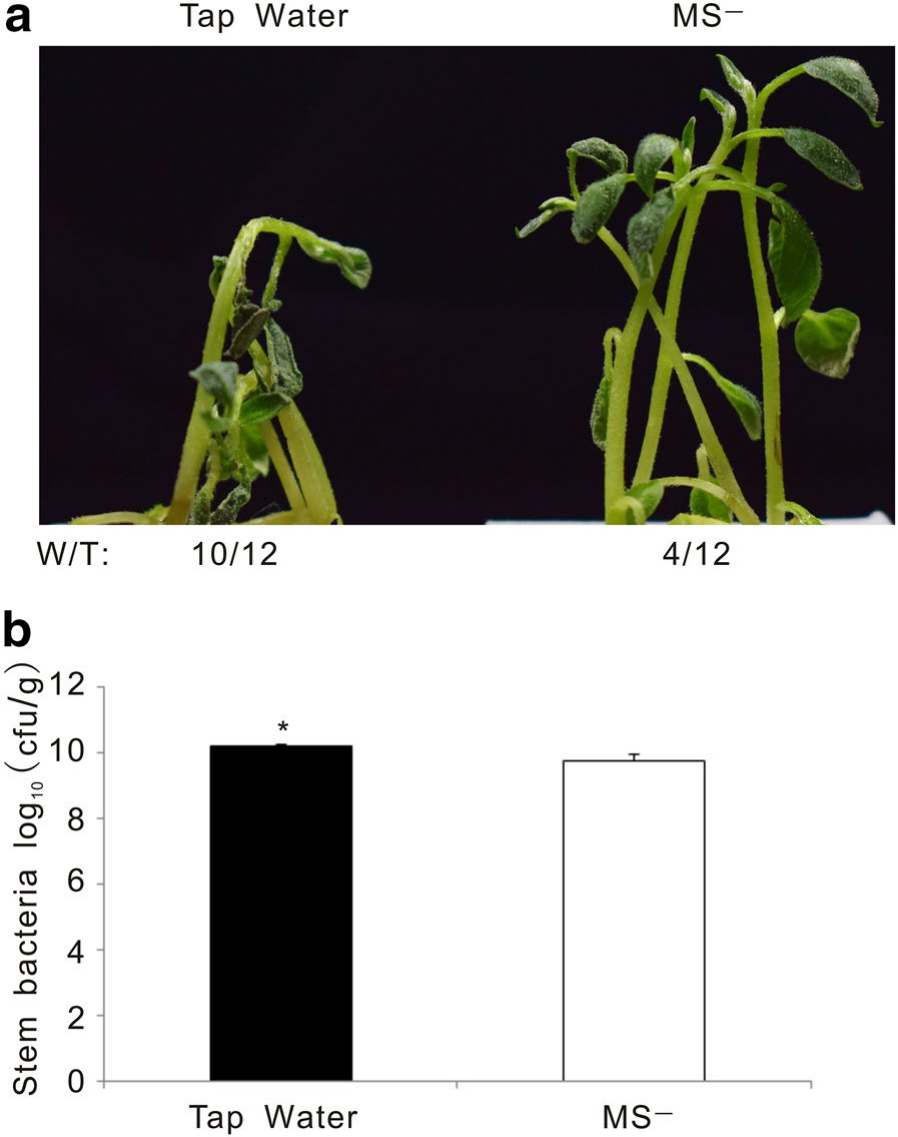
Tap water-resuspended bacterial solutions triggered stronger bacterial wilt disease symptoms than MS-resuspended bacterial solutions. **a** Wilting symptoms in representative photographs at 4 dpi. “W/T” represents the number of wilting plants with respect to the total of infected plants. **b** Bacterial content in potato stem recorded at 4 dpi. This experiment was repeated three times with similar results. Student’s *t* test was performed with respect to MS- solution (P < 0.05)

In the previous experiments, liquid MS medium containing many nutrients and vitamins (MS−) was used to re-suspend *R. solanacearum* for infection. To rule out a potential effect of the MS medium on *R. solanacearum* growth or virulence, we substituted the MS− medium with tap water. Wilting symptoms developed faster and stronger on potato plants in tap water containing *R. solanacearum* than in MS− medium with the same bacterial concentrations (Fig. 5a). In line with this observation, the amount of bacteria in potato plants treated with the water-resuspended pathogen was fivefold higher than in the potato plants treated with MS− resuspended bacteria (Fig. 5b).

### Hydroponic potato infection can be used to study *R. solanacearum* virulence in vitro

**Fig. 6:**
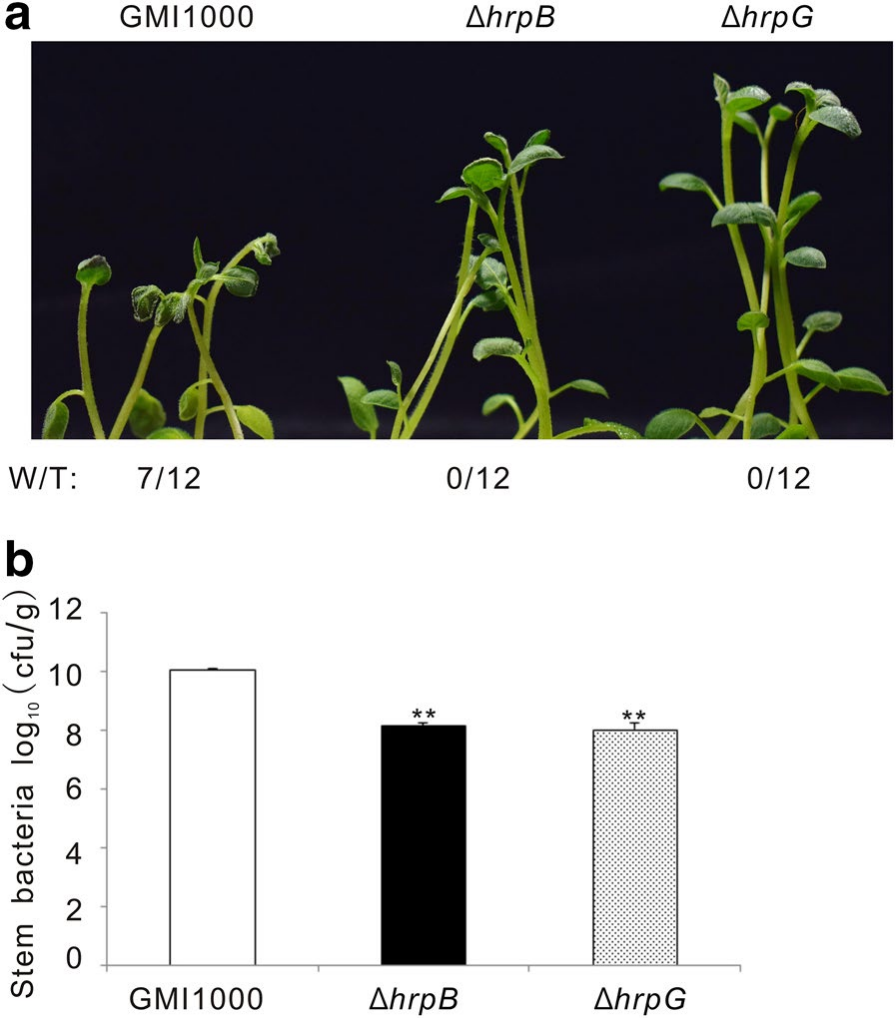
GMI1000 exhibited stronger virulence on potato than GMI1000(*ΔhrpB*) and GMI1000(*ΔhrpG*) mutants in our infection system. **a** Wilting symptoms observed in representative photos taken at 4 dpi. “W/T” represents the number of wilting plants with respect to the total of infected plants. **b** The bacterial content in potato stems recorded at 4 dpi. This experiment was repeated twice with similar results. **P < 0.001 (Student’s *t* test) with respect to GMI1000

**Fig. 7:**
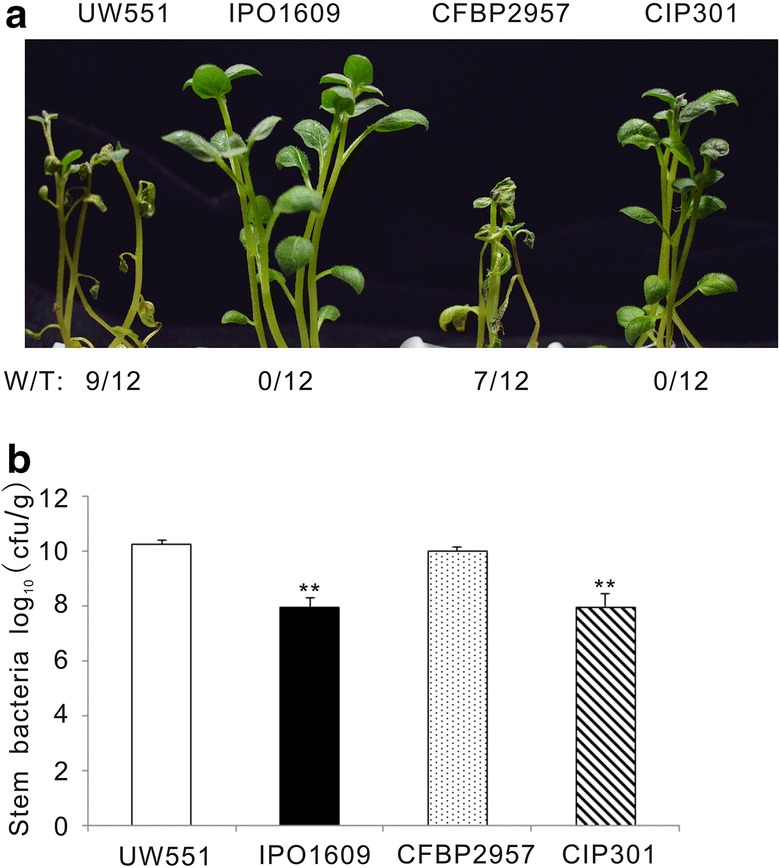
*R. solanacearum* strains showed differential virulence in in vitro potato infection system. **a** Representative picture of inoculated plants at 4 dpi. “W/T” indicates the number of wilting plants with respect to the total of infected plants. **b** The bacterial content in potato stems measured at 4 dpi. This experiment was repeated three times with similar results. **P < 0.001 (Student’s *t* test) comparing with UW551 strain

HrpG and HrpB, two key regulators of the bacterial type three secretion system, play critical regulatory roles in *R. solanacearum* virulence. The *hrpG* and *hrpB* mutant strains lose the ability to invade tomato [14, 23, 24]. To determine whether our in vitro infection system was suitable to evaluate *R. solanacearum* pathogenesis, we first infected potato plants with *R. solanacearum* GMI1000 wild type (wt), and the same strain carrying a precise deletion of the *hrpG* (*ΔhrpG*) or the *hrpB* coding sequence (*ΔhrpB*). The *hrpB* and *hrpG* mutants did not cause any bacterial wilt disease when compared with the strong wilting symptoms caused on potato plants by the wild type strain (Fig. 6a). Bacterial growth analysis showed that the potato colonization was similar in *hrpG* and *hrpB* mutants, but this bacterial content in stems was 100 fold lower than that of the wild type GMI1000 (Fig. 6b). These data indicate that mutations on *HrpB* and *HrpG* abolish *R. solanacearum* virulence on potato, which is consistent with the fact that both mutants are non-pathogenic on tomato and Arabidopsis [23, 25]. Next, we selected wild type *R. solanacearum* strains different from GMI1000 to test their aggressivity in our hydroponic potato infection system. Brown rot of potato is most commonly caused in the field by a subgroup of *R. solanacearum* strains belonging to phylotype IIB [26]. UW551 and IPO1609 from this group and the related CFBP2957 and CIP301 strains from phylotype IIA, were selected to investigate their virulence [6]. UW551 and CFBP2957 infection resulted in a strong leaf wilt symptom while IPO1609 and CIP301 did not cause any visible symptom (Fig. 7a). In addition, the multiplication of UW551 and CFBP2957 in potato was 100 fold higher than that of IPO1609 and CIP301 (Fig. 7b). Our results indicate that UW551 and CFBP2957 are much more aggressive on potato than IPO1609 and CIP301. Hence the in vitro infection system we have established here can be used to measure differences in the aggressivity of *R. solanacearum* stains on potato.

### Evaluation of the resistance of potato varieties to *R. solanacearum*

**Fig. 8:**
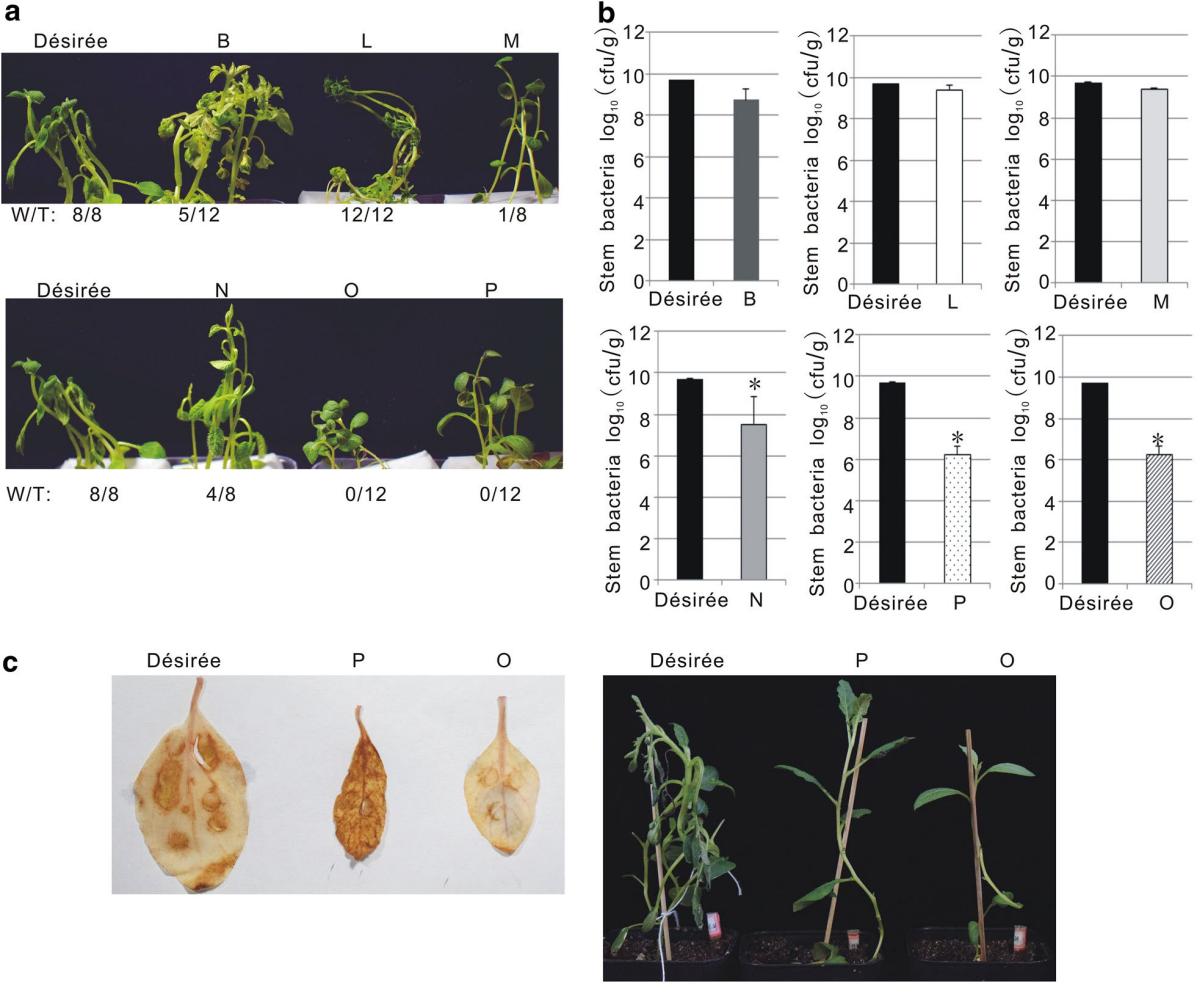
Potato varieties exhibited differential resistance to GMI1000 in the in vitro infection system. **a** Representative image of inoculated plants taken at 4 dpi. “W/T” represents the number of wilted plants over the total of infected plants. Tested varieties include: *Solanum tuberosum L.* Désirée; *S. tuberosum* subsp. andigenum (varieties B and N); *S. raphanifolium (varieties* M, O, P) and *S. pinnatisectum* (variety L). **b** Bacterial content in potato stems measured at 4 dpi. *P < 0.01 (Student’s *t* test) with respect to Désirée. **c** ROS production in the infiltrated leaves of potatos measured at 3 dpi. Left: DAB staining; Right: representative image of the plants for DAB staining assay taken at 6 dpi. These experiments were repeated at least twice with similar results

Easy identification of potato varieties with high resistance to *R. solanacearum* is a prerequisite for potato breeding programs. Hence, we performed a small scale experiment to test if our pathosystem could be used to efficiently screen for potato resistance to *R. solanacearum*. Thirty-two varieties of *Solanum tuberosum *L.* S. tuberosum* subsp. andigenum, *S. raphanifolium* and *S. pinnatisectum* were grown and inoculated using our hydroponic conditions. The results obtained from six of these varieties and the control susceptible variety Desirée are shown in Fig. 8. Wilting symptoms were clearly abolished on varieties O and P, and delayed on B, M and N species, compared to variety L and the control susceptible variety Désirée (Fig. 8a). Consistent with this, the population of the pathogen in O and P was 1000 fold lower than that in Désirée, suggesting O and P are highly resistant to *R. solanacearum* (Fig. 8). Interestingly, the pathogen population in M was twofold lower than that in Désirée (Fig. 8b), but significantly delayed wilting symptom development, indicating that M is a tolerant potato variety towards strain GMI1000. Reactive oxygen species (ROS) play a key role in plant defense [27]. To ascertain whether ROS production was triggered in the resistance plants O and P in response to *R. solanacearum*, we measured it in leaves of O, P and Desirée infiltrated with the pathogen. Comparing with Desirée and O, higher ROS level was detected in P plants at 3 dpi (Fig. 8c). This suggests that ROS signaling may be part of the defense responses of P leading to resistance to *R. solanacearum*. Moreover, while the leaf-infiltrated Desirée plants exhibited wilting symptoms, O and P plants did not (Fig. 8c). This indicates that the resistance of O against *R. solanacearum* seems to be controlled by alternative, ROS-independent mechanisms.

**Fig. 9:**
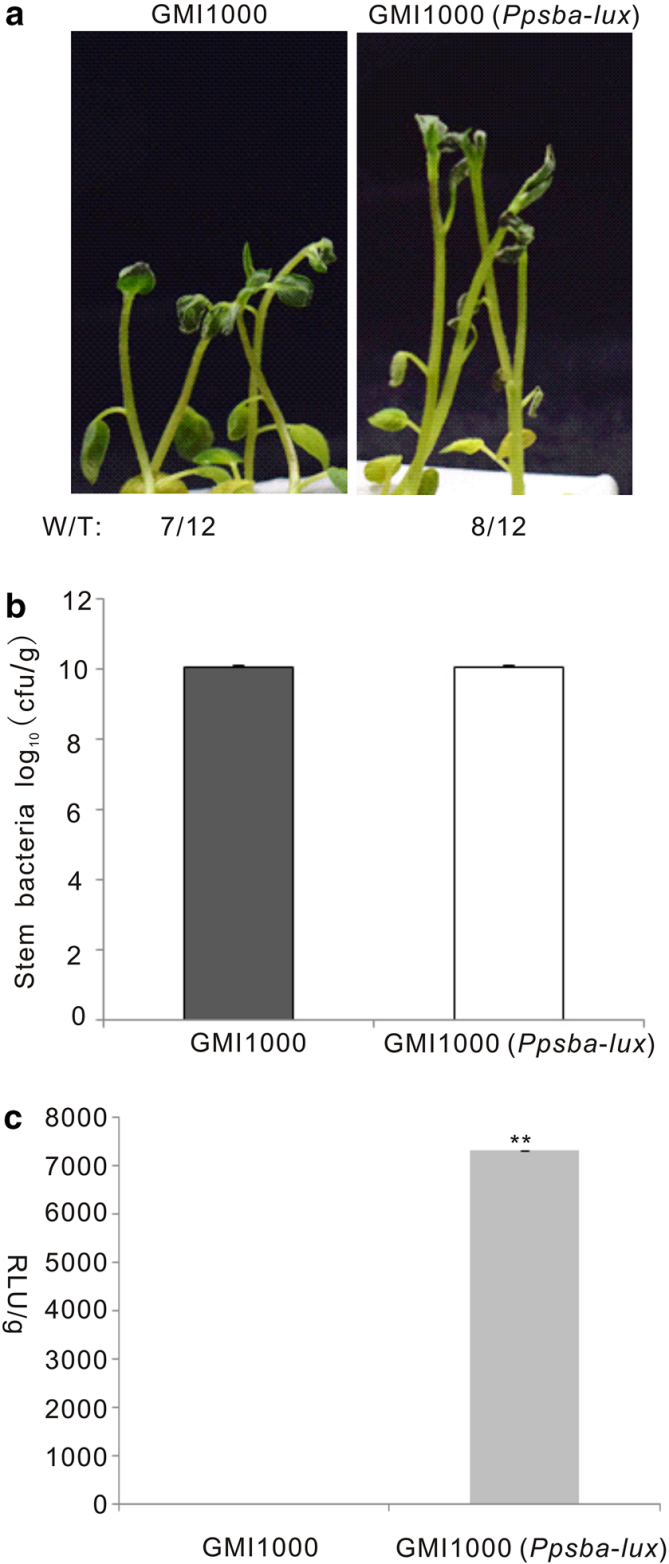
The growth of luminescent *R. solanacearum* was easily detected in plants. **a** Representative picture of infected potato plants taken at 5 dpi. **b** Bacterial content in the stem counted at 5 dpi. **c** Luminescence of *R. solanacearum* (*Ppsba-lux*) detected with a 96-well plate reader using the luminometer mode. This experiment was performed at least twice with similar results. **P < 0.001 (Student’s *t* test) with respect to GMI1000

The observation of symptomless plants does not always correlate with potato resistance to *R. solanacearum* as symptomless latent infections often occur [20]. Thus, bacterial counts must be also assayed. However, measuring bacteria in plants is a laborious task that cannot be applied at high scale to screen germplasm for resistance to *R. solanacearum*. Stable insertion of a *luxCDABE* luminescence reporter operon in the *R. solanacearum* genome has facilitated real-time monitoring of bacterial growth in plant hosts and has been used to evaluate potato resistance in plants grown in pots [20, 28]. To improve this screening method, we applied the *luxCDABE* luminescence reporter in our in vitro infection system. To this end, we used the strain GMI1000 (*Pps-Lux*) carrying the entire *Lux* operon under the control of the *PpsbA* chloroplast promoter, which exhibits strong, constitutive expression when introduced into *R. solanacearum* [20, 29]. As expected, potato plants infected with either GMI1000 or GMI1000 (*Ppsba-lux*) showed comparable wilting symptoms (Fig. 9a) and bacterial counts (Fig. 9b) in potato at 5 dpi. In addition, light detection with a luminometer showed a strong luminescence signal only in plants infected with GMI1000 (*Ppsba-Lux*) (Fig. 9c). These data corroborate that insertion of the *PpsbA::luxCDABE* in the *R. solanacearum* genome affect neither its colonization ability nor its capacity to cause disease symptoms. The application of a luminescence reporter increases the efficiency of our hydroponic potato infection system for evaluation of potato germplasm resistance to bacterial wilt.

## Discussion

The interaction between *R. solanacearum* and its plant hosts has been established as a model system to study plant resistance to soil-borne bacterial phytopathogens for more than two decades [10, 24]. Soil-drench and/or stem penetration inoculations are mostly used to investigate bacterial wilt disease progress on tomato, eggplant, potato, the model plants *Medicago truncatula* and Arabidopsis [10-13, 20, 30]. Using any of these two infection methods requires a large amount of time and space, with the ensuing high costs. Moreover, the growth in soil prevents the investigation of early root responses to the pathogen [17, 20]. To overcome these problems, we set up here an in vitro inoculation assay on potato. The in vitro potato inoculations have previously been used to quantify blackleg disease on shoots, showing results comparable to greenhouse assays [31]. Chen and colleagues successfully identified three SSR alleles related to bacterial wilt resistance from *Solanum tuberosum* + *S. chacoense* somatic hybrids through in vitro inoculation of potato plants grown in solid medium [32]. However, their infection protocol was not described explicitly. Here, we thoroughly described a quick, accurate and space-saving potato infection system to monitor *R. solanacearum* using plants grown under hydroponic in vitro conditions. In our system, four potato plants were directly propagated into a container and infected two weeks later with a *R. solanacearum* solution. Compared to soil drench inoculation [20] our method saves two-weeks. In our assay, 75% of plants were completely wilted at 2–3 days after the first wilting symptoms were recorded (Figs. 2a, 4a, 5a and 8a), showing that this assay is very stable and repetitive. Our recently established in vitro infection system for Arabidopsis grown on agar plates has shown that *R. solanacearum* infection changes the root architecture [16, 17, 33]. This phenomenon could not be observed and investigated by means of a traditional soil-drench or stem penetration inoculations. Thus, our assay provides the possibility to investigate early potato responses to *R. solanacearum*.

The HrpG and HrpB transcriptional regulators control the virulence of *R. solanacearum* through modulating the expression of the genes encoding the type three secretion system and its related effectors [25, 34]. The deletion of *hrpG* and *hrpB* abolished wilt symptom occurrence and restrained the pathogen proliferation in potato plants (Fig. 6), which is consistent with the mutant strains loss ability to infect on the tomato and Arabidopsis [14, 17]. However, while the *△hrpG* deletion mutant grew more than *△hrpB* in tomato stems [14], these two strains grew to similar levels in potato. This could be a host species-dependent phenomenon. In line with this hypothesis, the capacity of the *△hrpB* strain to colonize Arabidopsis seems to be stronger than that of the *△hrpG* strain [17].

*R. solanacearum* strains UW551 and IPO1609 belong to race 3 biovar 2, which causes potato brown rot at cool temperatures [26]. In our *potato* infection assay UW551 was much more aggressive than IPO1609, causing stronger wilting symptoms and increased bacterial growth. In accordance with this, it has been reported that the pathogenicity of IPO1609 was strongly attenuated on tomato and potato relative to UW551 when using a soil-drench inoculation method, due to a major deletion present in its genome [26]. We also found that strain CIP301, isolated from potato, did not display strong virulence on potato. Therefore we speculate it may be a hypoaggressive strain similar to IPO1609. CFBP2957 from tomato exhibited hypervirulence on potato in our assay. This is not surprising, as it has been known for long that host range in nature does not always correlate with aggressivity on different hosts under laboratory conditions. For instance, UW551, a potato strain, has been reported to cause strong bacterial wilt on tomato [26]. All these data indicate that this in vitro infection assay is suitable for evaluating the pathogenicity of *R. solanacearum* strains on potato as accurately as when soil drench inoculation is used. In addition, our hydroponic infection also provides the possibility to directly investigate the interaction between potato root and other soil-borne pathogens.

Three wild type potato lines were identified with higher bacterial wilt resistance among 32 tested candidate lines. This indicates that the in vitro infection system established here can be effectively applied to high-throughput screening for bacterial wilt resistance in potato germplasm. Wilting symptoms are the simplest way to evaluate plant resistance to bacterial wilt. However, symptom recording is time consuming and latent symptomless infections that can cause havoc when environmental conditions change [20, 35, 36] escape detection. Thus, latent infection limits the application of leaf wilting to evaluate potato resistance to the pathogen. To overcome this problem, we employed a luminescent reporter strain [20, 29] in our infection system to be able to quantify bacteria inside the plant, which may not have caused symptoms. Luminescence intensity was positively correlated with bacteria colonization in the infected plant stem (Fig. 9). However, unlike our previous studies [20], bacterial colonization in the infected plant could not be visualized in this work using a light imaging system (ChemiDoc ™ XRST). One reason for this could that the luminescent GMI1000 strain originally isolated from tomato is less aggressive than the luminescent UY031 strain on potato that we used in previous reports [37, 38]. In addition, it is possible that the bacterial concentrations carried by the younger plants used here are below the detection limits of the light imaging system. In any case, we could effectively quantify the luminescent bacteria with a luminometer. Compared with colony counting after dilution plating, detection of bacterial luminescence from crushed stems using a 96-well plate luminometer is a faster, more reliable procedure.

## Conclusion

In this study, a hydroponic potato infection assay in vitro has successfully been established for *R. solanacearum*. This assay is less time-consuming, low-cost, accurate and easier to handle comparing with the previously described and widely used infection assays. We demonstrated that it can also be applicable for large-scale screening of potato germplasm for resistance to brown rot disease, which will speed up and increase the efficiency of breeding resistance into potato cultivars.

## Materials and methods

### Plants and strains

Two centimeter shoot explants from *Solanum tuberosum *L. Désirée; B and N from *S. tuberosum* subsp. Andigenum; M, O, P from *S. Raphanifolium*; L from *S. Pinnatisectum*) were cut and inserted into paper holders which were immersed in 35 ml MS liquid medium (4.405 g/l MS salt including vitamins, 20 g/l sucrose, pH 5.8). Four plants were grown in each glass jar (diameter = 8 cm), containing 35 ml of MS- solution (or tap water after infection when indicated). Plants were grown in a chamber under long day conditions (16 h light, 8 h dark), 23 °C, 75% humidity and 10,000 lx light intensity conditions.

To prepare bacterial inocula, 2–3 single *R. solanacearum* colonies (strains GMI1000, UW551, IPO1609, CFBP2957 or CIP301) were transferred into 10 ml liquid B medium (10 g/l peptone, 1 g/l yeast extract and 1 g/l casamino acid) and incubated overnight at 28 °C in a shaker.

### In vitro potato infection assay

Overnight *R. solanacearum* cultures were collected by centrifugation (4000 rpm, 5 min), washed once with MS-/tap water, diluted with MS−/tap water and adjusted to OD_600_ = 0.01. Then the bacterial suspensions were distributed into jars, using 35 ml per jar for infection.

Roots of 2-week-old potato plants were cut with scissors 2 cm below the stem and put into the bacterial suspension for inoculation. Inoculated potato plants were kept in the growth chamber under long day conditions (16 h light, 8 h dark), 25 °C and 10,000 lx light and 70% humidity. At 2 dpi, the lid of the jar containing the infected plants was loosened to allow air exchange. Wilting symptoms on the infected plants were recorded by taking digital images at the indicated times.

### DAB staining assay

Plant leaves were directly infiltrated with *R. solanacearum* solution at OD = 0.001. Infiltrated leaves were detached at the indicated time and immediately immersed into 1 mg/ml DAB solution for overnight in the dark. Then the leaves were de-stained with absolute ethanol and boiled for 10 min and photographed.

### Bacteria counting and bacteria luminescence quantification

The aerial part of the infected plants was harvested 1 cm above the level of the liquid in the jars and weighed, then homogenized with pestle and mortar. Two ml double distilled water (ddH_2_O) was added and mixed with the plant material and the homogenates were serially diluted in water and plated on solid B medium. Plates were kept in the 28 °C incubator for 48 h and bacterial colonies were counted. The bacterial contents in the stem (cfu/fresh weight of aerial part of the infected plants) was used to evaluate bacterial virulence or plant resistance.

For luminescence measurement assays, the homogenates from the aerial tissues of infected plants were transferred to a 96-well plate (Nunclone) and the luminescence emitted from the pathogen was measured and quantified with a plate reader infinite 200 Pro (Tecan). Luminescence readings were normalized to the fresh weight of each sample and presented as RLU (relative luminescence units) per gram of fresh tissue.

## Data Availability

All data generated or analysed during the this study are included in this published article.
